# A Systematic Review of the Burden of Stroke in Ghana

**DOI:** 10.1155/2024/8298154

**Published:** 2024-10-05

**Authors:** Joseph Attakorah, Kofi Boamah Mensah, Peter Yamoah, Ebenezer Wiafe, Varsha Bangalee, Frasia Oosthuizen

**Affiliations:** ^1^Directorate of Internal Medicine, Komfo Anokye Teaching Hospital, Box 1934, Kumasi, Ghana; ^2^Department of Pharmacy Practice, College of Health Sciences, Kwame Nkrumah University of Science and Technology, Kumasi, Ghana; ^3^Discipline of Pharmaceutical Sciences, College of Health Sciences, University of KwaZulu-Natal, Westville Campus, University Road, Durban, South Africa; ^4^School of Pharmacy, University of Health and Allied Sciences, Volta Region, Ghana

**Keywords:** burden, Ghana, incidence, meta-analysis, prevalence, stroke

## Abstract

**Background:** Stroke is considered a significant public health concern in sub-Saharan Africa and Ghana due to its impact on quality of life. However, there is a lack of comprehensive pooled data on the prevalence and incidence rates of stroke in Ghana. Updating this information would help inform decision-making bodies on measures to reduce the burden of stroke in Ghana. This systematic review is aimed at critically appraising evidence gathered from studies done in Ghana on the prevalence and incidence rates of stroke among the Ghanaian population.

**Method:** Four databases (CINAHL via EBSCOhost, Web of Science, MEDLINE via PubMed, and PsycINFO via EBSCOhost) were searched, for articles published between May 2000 and May 2020 on stroke burden. The search was constrained to studies conducted in Ghana and published in English that have been peer reviewed. Cochrane risk of bias tool was used to assess the quality of evidence. Meta-analysis was conducted to estimate the pooled stroke prevalence and incidence in the country.

**Results:** A total of three studies that documented 12,974 stroke cases in 1,197,498 participants based on the inclusion criteria were reviewed. The meta-analysis revealed that the overall national prevalence and incidence rate of stroke for the country were 7.96% and 1.17%, respectively, calculated at 95% confidence intervals.

**Conclusion:** According to the review findings, the incidence and prevalence rates of stroke are high in Ghana or among the Ghanaian population, and they are increasing.

## 1. Introduction

Globally, stroke, a neurological condition, is the second leading cause of death worldwide [1]. The 2019 Global Burden of Diseases (GBD) study also revealed that stroke was the second leading cause of death and the third leading cause of death and disability worldwide [2]. The burden has increased due to people's lifestyle and aging, causing a lot of economic pressure on healthcare systems globally.

In 2019, the age-standardized stroke-related mortality rate was 36 (35–38) times higher in the developing countries than in the developed countries, while the age-standardized stroke-related disability-adjusted life year (DALY) rate was 37 (35–39) times higher [2]. For the past two decades, many studies have been conducted and published that covered the issue of stroke in sub-Saharan Africa and Ghana on different topics [3–13]. A study conducted by Akpalu et al. [3] examined the outcome and determinants of stroke in patients with and without diabetes at a tertiary hospital in Ghana [3]. Donkor et al. [6] assessed the level of awareness about stroke in Accra, the capital city of Ghana. Findings of few studies that provide insights into the prevalence and incidence of stroke in Ghana could not be generalized by each study, as the researches were conducted only in some specific regions in the country leaving a knowledge gap on what the real burden on the country really looks like [9–11, 14].

There is also lack of reliable reporting mechanisms and disease or death registration systems in Ghana [15, 16], so the epidemiological findings from the GBD study are likely to be underestimated. In the past two decades, Ghana has experienced significant demographic, economic, and epidemiological change [17, 18]. These factors have led to an increase in life expectancy and, consequently, an increase in the population's age structure [19]. There are very few reliable morbidity and mortality estimates for stroke in Ghana [14]. Moreover, the available research on the epidemiology of stroke in Ghana contains a number of methodological flaws, such as small and variable sample sizes, inconsistent diagnostic criteria, different case definitions, and survey strategies. The majority of these studies are cross-sectional, and their primary aims are also diverse.

Understanding the epidemiology of stroke in Ghana is difficult due to a lack of data and reliable reporting mechanisms. In order to investigate the incidence and prevalence of stroke in Ghana and to comprehend its true scope, we conducted a systematic review of epidemiological studies of stroke in Ghana.

## 2. Methods

The authors followed the structured guidelines for systematic reviews found in the Cochrane Handbook [20, 21].

### 2.1. Eligibility Criteria

The review included any population-based, cross-sectional, and cohort studies that looked into the prevalence and incidence of stroke in Ghana. Studies were eligible upon meeting few predefined criteria. Firstly, articles that were published in a stroke journal between the period of May 2000 and May 2020: studies published before or after the duration were excluded. Secondly, articles that are in full-length and published in English: thus, all other publications, such as research notes, editors' comments, conference abstract, readers' comments, and book reviews were excluded. Finally, studies that are related to stroke prevalence and incidence: unrelated studies were considered irrelevant articles and thus excluded.

### 2.2. Information Sources

An extensive literature search was conducted manually using four different databases namely CINAHL (EBSCOhost), MEDLINE (PubMed), PsycINFO (EBSCOhost), and Web of Science by the reviewer to establish evidence.

### 2.3. Search Strategy

Two independent reviewers KBM and JA screened the title or abstract of all citations or selected articles based on relevance and the set criteria. Any disagreements were resolved by recourse to a third reviewer (PY) to reach a consensus. When there were multiple papers from the same research population with indications of overlapping data or identical participant characteristics, the article with the largest dataset was chosen. Collection of literature was done between March and June 2019, and additional literature search was conducted in February to May 2022. The systematic review was designed and carried out following recommendations from the Preferred Reporting items for Systematic Review and Meta-Analysis (PRISMA) protocols. The protocol was registered with OSF with registration number [22]. Citations and articles from the reference list of the identified stroke articles were also traced and reviewed. The search strategy used search terms based on Medical Subject Heading (MeSH) terms. The following search terms were used: isch(a) emic stroke, stroke, intracerebral, subarachnoid, intraparenchymal, haemorrhage, incidence, epidemiology, attack rates, survey, surveillance,case-fatality, mortality, morbidity, fatality, and trends. These searches were done using the AND/OR Boolean logic operator.

### 2.4. Study Selection Process

**Figure 1:**
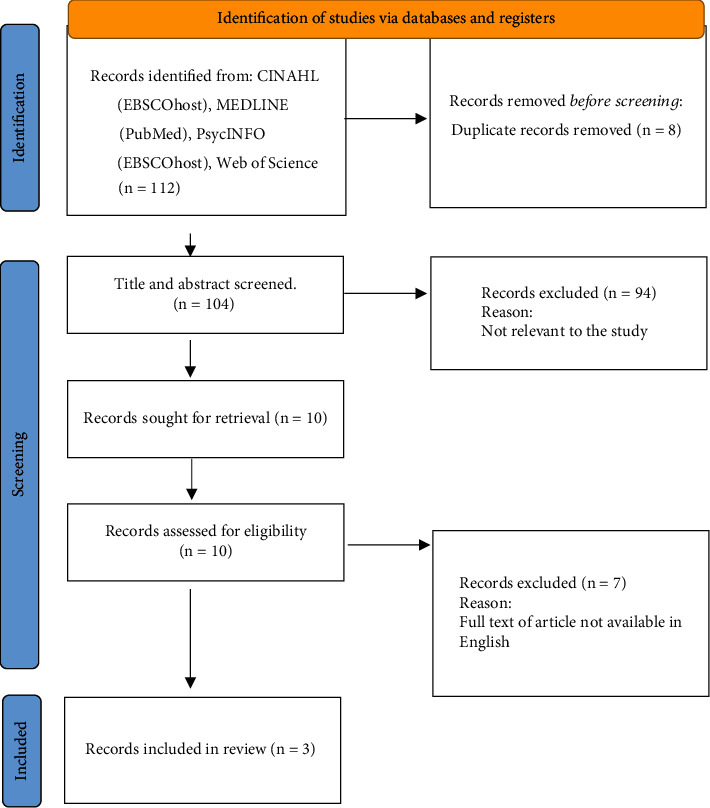
Summary of study selection process using the PRISMA 2020 flow chat.

A total of 112 publications were identified, and only those empirical studies related to the stroke prevalence and incidence were selected for the analysis. A total of eight duplicate records were removed prior to the screening. After screening, three studies met the criteria for inclusion in the review. The flowchart illustrating the study selection procedure in accordance with the PRISMA statement is shown in Figure 1.

### 2.5. Data Collection and Analysis

Data from the included studies were extracted by the primary reviewer (JA) and the second reviewer (KBM) using a standardized research matrix and verified by a third reviewer (PY). Any difference or disagreements noticed were resolved in a well-organized discussion by the team to reach a consensus. The pooled prevalence of stroke and its 95% Cl were estimated using STATA/SE (version 15.0; Stata Corp LLC, College Station, Texas, United States). The overall prevalence and incidence were estimated by meta-analysis.

## 3. Results

A total of 112 records were found through the search of which 104 records were found for screening after duplicates were removed. Ten records were chosen after independent screening that qualified for full-text review. Three population-based cross-sectional studies were found to meet our inclusion criteria after reading the full texts of these 10 records. The remaining seven were not eligible for inclusion because the full text of the articles was not available in English.

### 3.1. Overall Stroke Prevalence and Incidence in Ghana

This study included a total of three studies that documented 12,974 stroke cases in 1,197,498 admissions. The overall estimate of the national prevalence and incidence of stroke based on the three studies was 7.96% and 1.17%, respectively, calculated at 95% confidence intervals. The individual studies reported different prevalence and incidences of stroke based on the location and recruited participants that took part in each study. The detailed information of the prevalence and incidence rates are provided in Table 1.

### 3.2. Study Characteristics

The characteristics of the three studies are shown in Table 2. The studies were published from 2015 to 2019 and were conducted in the following regions in Ghana, namely, Ashanti, northern, eastern, western, central, Bono Ahafo, Volta, upper west, upper east, and Greater Accra. The disease state of some of the study populations was identified as hypertension and diabetes. The gender distribution of the study population was presented in only one study [10]. Prevalence, incidence, and mortality of stroke were discussed in all the selected studies. Two of the selected studies employed a prospective study design [10, 11] while the other employed a retrospective design [9].

### 3.3. Summary of Study Outcome

The first study assessed the trends in stroke admission and mortality rates from 1983 to 2013 in central Ghana [9]. This study found that the admission rate for stroke increased from 5.32 per 1000 hospital admissions in 1983 to 13.59 per 1000 hospital admissions in 2013. The mortality rate due to stroke increased from 3.4% per 1000 deaths in general hospitals in 1983 to 7.6% per 1000 deaths in general hospitals in 2013. The fatality rate ranged between 32.5% and 49.0% during the duration of the study. The second study titled “Incident stroke among Ghanaians with hypertension and diabetes: A multicentre prospective cohort study” [10] also reported that overall, the incidence rate of stroke was 14.19 per 1000 person-years (95% CI: 10.77–18.38). Hence, stroke incidence is extremely prevalent among Ghanaians receiving treatment in public facilities for hypertension and diabetes. The last study reviewed concluded that stroke incidence rates (95% CI) increased with decreasing eGFR categories of 89, 60–88, 30–59, and 29 mL/min, corresponding to incidence rates of 7.58 (3.58–13.51), 14.45 (9.07–21.92), 29.43 (15.95–50.04), and 66.23 (16.85–180.20) per 1000 person-years, respectively. Details are presented in Table 3.

### 3.4. Quality Assessment of Selected Studies

For assessment of the included studies, a quality assessment tool proposed by Attakorah et al. [22] was used to assess the quality of the selected studies. Based on the quality assessment criteria, the studies were scored. An assessment scores between 0% and 33.9% was regarded as weak, 34% and 66.9% as moderate, and 67% and 100% as strong. All the studies were reported as having a strong methodological quality.

### 3.5. Assessment of Risk of Bias

The WHO STEPwise approach to stroke surveillance (WHO STEPS) is a framework designed to provide standardized methods for collecting, analyzing, and disseminating data on stroke. It emphasizes three key steps: assessing risk factors (Step 1), physical measurements (Step 2), and biochemical measurements (Step 3). When evaluating the risk of bias in a study using the WHO STEPS framework, it's essential to consider several aspects: representativeness, measurement accuracy, potential confounders, and data collection methods.

The studies included in this study defined stroke using the World Health Organization (WHO) criteria and were guided by the WHO STEPS framework. The articles were rated as having low risks of bias based on stipulated criteria. The details are provided in Table 4.

### 3.6. Sensitivity or Subgroup Analyses

The systematic review conducted on the burden of stroke in Ghana included subgroup analyses to better understand the impact of various demographic and clinical factors on stroke incidence and prevalence. Specifically, one study within the review stratified stroke incidence by age, revealing that the incidence rates were markedly higher in older age groups, particularly among those aged 50–69 years, compared to younger cohorts. Additionally, subgroup analyses by comorbid conditions, such as hypertension and diabetes, were also performed. These analyses demonstrated that the incidence of stroke was significantly higher among individuals with both hypertension and diabetes compared to those with only one or neither of these conditions.

Moreover, the analysis considered regional differences, highlighting variations in stroke prevalence and incidence across different regions of Ghana. These variations suggest potential differences in healthcare access, lifestyle factors, or genetic predispositions that could contribute to the observed disparities in stroke burden. However, it is important to note that the limited number of studies included in the meta-analysis and the heterogeneity in study designs, sample sizes, and diagnostic criteria may affect the robustness of these subgroup analyses.

The results underscore the importance of tailored public health interventions that address the specific needs of high-risk subgroups, such as the elderly and those with comorbidities, to effectively reduce the stroke burden in Ghana.

## 4. Discussion

The current study is aimed at estimating the prevalence and incidence rates of stroke among the populace in Ghana using systematic literature review and meta-analysis. After combining the data gathered from the three articles included, that documented 12,974 stroke cases in 1,197,498 admissions and analyzing the data using meta-analysis, the overall prevalence and incidence were estimated to be 7.96% and 1.17%, respectively. This suggests that generally, the burden of stroke is high in Ghana. These outcomes may be due to several factors such as lack of awareness of stroke and its risk factors (hypertension and diabetes), lifestyle, insufficient health infrastructure, inadequate medical equipment (CT and MRI scanners) at stroke treatment centers, inadequate skilled personnel, and physicians. This was also reported by Sarfo et al., in their study [8, 9]. Similar reports have also been reported in Benin [23] and Nigeria [24]. Some studies done outside Africa have stated poor community knowledge of stroke risk factors, and this is evident in the data from Brazil [25], Ireland [26], Australia [27], Pakistan [28], and the United States [29]. In contrast, one study conducted among university staff and students in Nigeria reported an appreciable level of awareness of stroke risk factors with the majority of the respondents correctly identifying each of the major stroke risk factors [30]. This might be due to the fact that because they are in the university and with their educational background, they might have been exposed to seminars or discussions relating to stroke, thus being knowledgeable.

It is evident from this study that the general burden of stroke in Ghana is not known owing to lack of extensive research and publications on stroke. Poor awareness of stroke and its risk factors has been reported to be one of the key contributors to the burden of stroke in low- and middle-income countries [8, 9]. Stroke is a serious condition that requires emergency treatment; hence, there is a need for increased awareness, extensive research, and education about stroke as well as a need for improvement in healthcare resources and infrastructure to address the growing burden of stroke. It is worth noting that evidence suggests that many patients and caregivers continue to express a lack of understanding about stroke and its causes, secondary preventative measures, and information about both statutory and informal support [31]. Another study conducted has shown that increased awareness of stroke risk factors among people at high risk for stroke leads to improved compliance with stroke prevention practices [32]. In Ghana, there is currently a stroke epidemic, which could partly reflect the poor awareness of stroke risk factors observed in a study conducted in Ghana [33]. The stroke burden however varied by regions in each individual study (21.54%, 1.71%) especially in areas where hypertension and diabetes were very common among the participants as reported by [11].

Lack of stroke education can also lead to increased hospital stroke admissions and stroke mortality. A study conducted by Agyemang et al. [34] showed that stroke constitutes a considerable cause of morbidity and mortality in Ghana. A similar study conducted in South Africa (published in 2012) also showed high stroke admission rate [29] and high fatality rate [35]. This high fatality rate may be due to the severe scarcity and prohibitive costs of facilities and human resources for investigations, acute care, and rehabilitation of stroke patients in Africa.

Other key findings of Sarfo et al. [10] revealed increasing stroke incidence with aging population with comorbidities such as hypertension and diabetes. With an increase in the aging population in the world, the global burden of stroke is set to increase, which is reported by Sanuade et al., where increase in stroke prevalence correlates with aging populations living with hypertension or diabetes [14]. The increasing prevalence of stroke in Ghana is likely to put a huge pressure on already weak healthcare system and threaten the viability of poorly funded public health and primary healthcare services [36]. The increasing burden of stroke also has huge economic implications, especially given that nearly half of the stroke patients were below 65 years of age [10]. The economic impact of stroke could be felt as both cost to the country's healthcare system and the loss of income and production of those affected either directly by the disease or indirectly as caregivers to those with stroke.

Our findings underscore the urgent need for both clinical and public health measures to minimize the burden of stroke in Ghana. In view of the scarcity of resources in Ghana, the activities are aimed at controlling stroke and other cardiovascular diseases (CVAs) will have to compete with many other pressing health needs [37]. Nevertheless, the burden of stroke in Ghana is now evident and substantial and can no longer be swept under the carpet.

In addition, the incidence of stroke is on the rise. As life expectancy increases in Ghana, the number of people who will experience a stroke will increase significantly soon. It is therefore imperative that urgent measures be taken to reduce the risks and thereby optimize the health outcomes for stroke and other CVDs in Ghana. Stroke prevention is best accomplished by the control of risk factors, particularly hypertension [38]. This will require a multidisciplinary approach and sustained effort involving a broad range of interventions and resources [33]. A strong political commitment is also needed to promote the relevant policy and environmental changes to support adequate education and prevention programs in Ghana. The establishment of national guidelines for prevention, detection, treatment, and control of stroke and risk factors such as hypertension will be a tangible essential step [38]. These measures could have a considerable impact on reducing stroke and other CVDs in Ghana.

A national CVD prevention program in Mauritius showed substantial reductions in CVD risk factors [39]. One of the approaches that should be adopted by the healthcare workers in Ghana is to raise awareness through education regarding warning signs and risk factors of stroke. Therefore, much must be done to improve the knowledge of these warning signs and risk factors. It has been shown that there have not been many studies done to analyze the burden of stroke in prevalence, incidence, and mortality in Ghana for the past 20 years. Hence, there is a need for more studies to be conducted in this area to draw a better conclusion, which will inform policy. Policymakers, as well as local and national organizations, need sustained and intensified educational efforts to promote knowledge of stroke, particularly among high-risk groups especially elderly, and improve funding for health infrastructures, provision of medical instruments (CT scanner and MRI scans), and availability of skilled personnel to managed cerebrovascular disease in Ghana.

### 4.1. Limitations

Even though the process of systematic literature review and meta-analysis is known to be the best practical way of generating more powerful estimations, this study does come with some limitations. One of the limitations of this present study was the small number of studies conducted in Ghana, making the generalization of the results obtained from them on a large scale difficult. Thus, it is suggested that further studies with larger sample sizes across the country should be conducted to provide more data to clarify the stroke burden. Secondly, the search was limited to four databases and did not include data from grey literature. In addition, the search was restricted to studies conducted and published between May 2000 and May 2020 and studies published in English. Hence, there is a need for further studies on a larger scale in the region to improve these limitations.

## 5. Conclusion and Recommendation

Based on the findings of this current study, the prevalence and incidence rates of stroke is high in Ghana or among the Ghanaian populace and its rates are on the ascendancy. Therefore, it is recommended that health officials and policymakers pay more attention to the prevention and control of stroke in Ghana. Government of Ghana must also invest in the health and infrastructure of the country by procuring the equipment needed for proper functioning and delivery of stroke treatment centers. Population-based health education programs and appropriate public health policy also need to be developed.

## Figures and Tables

**Table 1:** Pooled stroke prevalence and incidence (%) in Ghana based on meta-analysis.

	**Study name**	**No. of participants enrolled**	**No. of participants with stroke**	**No. of participants without stroke**	**New stroke cases recorded**	**Incidence rate**	**Prevalence**	**OIR**	**OPR**
Study 1	[9]	1,190,906	12,233	1,178,673	1536	0.13	1.03	1.17	7.96
Study 2	[10]	3296	76	3220	54	1.68	1.30		
Study 3	[11]	3296	665	2631	45	1.71	21.54		

Abbreviations: OIR, overall incidence rate; OPR, overall prevalence rate.

**Table 2:** Characteristic of studies included in the review.

**Author and year of publication**	**Region(s) of the study in Ghana**	**Study objectives**	**Disease state of study population**	**Gender distribution of study population**	**Study design**	**Duration of study (months)**	**Sample size of study population**
[9]	Ashanti	To evaluate 30-year trends (1983–2013) in stroke admission and mortality rates in central Ghana.	Missing	Approximately equal gender distribution	Retrospective study	Missing	12,233
[10]	Ashanti, northern, eastern, Greater Accra	To evaluate the determinants of stroke among a prospective cohort of Ghanaians with hypertension and diabetes mellitus.	Hypertension, diabetes	Male = 776, female = 2520	Prospective cohort study	9	3296
[11]	Ashanti, northern, eastern, Greater Accra	Estimated glomerular filtration rate predicts incident stroke among Ghanaians with diabetes and hypertension.	Hypertension, diabetes, renal impairment	Missing	Prospective cohort study	18	3296

**Table 3:** Results of individual studies included in the review.

**Title of study**	**Study outcomes**	**Conclusion**	**Limitations**
1. Trends in stroke admission and mortality rates from 1983 to 2013 in central Ghana	There was a 260% increase in stroke admissions from the year 1983 to 2013. Stroke admission rate increased gradually from 5.32 per 1000 general hospital admissions in 1983 to 13.59 per 1000 general hospital admissions in 2013. A total of 1132 patients were adequately diagnosed and classified as intracerebral haemorrhage (569 [50.3%]), ischemic stroke (382 [33.7%]), and subarachnoid haemorrhage (181 [16.0%]). Stroke fatality increased from 3.4 per 1000 general hospital deaths in 1983 to 7.66 in 2013. The fatality rate for the study duration varied between 32.5% and 49.0%. Postadmission stroke–related deaths were 31.1%, 43.7%, 18.9%, 5.5%, and 0.9% at 24 h, 2–7 days, 8–14 days, 15–28 days, and over 28 days, respectively. In a 28-day in-patient admission, case fatality rate of 41.1% was estimated.	Over the duration of the data analysis on stroke admissions and fatality, the two variables have increased gradually. Prompt action is required to address these observed trends.	1. Unavailability of stroke data for the years 1989 and 1990 2. Lack of computerized tomography scanner (until 2008) and magnetic resonance imaging scanner (until 2011) at the study site 3. The sociodemographic features of study subjects, which could have explained the increased stroke mortality on admissions, were not considered 4. Comorbid medical conditions which could have contributed to stroke-related mortality were not studied

2. Incident stroke among Ghanaians with hypertension and diabetes: A multicentre prospective cohort study	Incidence rate of stroke overall was 14.19 events per 1000 person years (95% CI: 10.77–18.38). Incidence rate among age < 40 years was 3.48, 40–49 years was 9.50, 50–59 years was 15.57, 60–69 years was 21.41, 70–79 years was 12.82, and 80+ years was 16.00 per 1000 person-years. There were 5 strokes among 419 diabetics, 21 out of 985 diabetics with hypertension had strokes, and 28 strokes among 1816 participants with hypertension. Incidence rate of stroke among those with diabetes alone was 9.81 (3.59–21.74)/1000 person-years, hypertension alone was 13.77 (9.33–19.64)/1000 person-years, and among those with both diabetes with hypertension was 16.64 (10.58–25.00)/1000 person years.	Incident strokes are highly common among Ghanaians with hypertension and diabetes receiving treatment in public hospitals.	1. Lack of confirmation of stroke with neuroimaging 2. The study relied on clinical assessments by study physicians to confirm stroke diagnosis, which may be subject to misclassification by stroke mimics 3. Unavailability of CT scans at most of the study sites 4. There was a possibility that some severe or fatal stroke cases who did not reported to the clinic for follow-up were missed since strokes were assessed only among participants who reported for follow-up visits

3. Estimated glomerular filtration rate predicts incident stroke among Ghanaians with diabetes and hypertension	Stroke incidence rates (95% CI) increased with decreasing eGFR categories of 89, 60–88, 30–59, and < 29 mL/min corresponding to incidence rates of 7.58 (3.58–13.51), 14.45 (9.07–21.92), 29.43 (15.95–50.04), and 66.23 (16.85–180.20) per 1000 person-years, respectively. Adjusted hazard ratios (95% CI) for stroke occurrence according to eGFR were 1.42 (0.63–3.21) for eGFR of 60-89 mL/min, 1.88 (1.17–3.02) for 30–59 mL/min, and 1.52 (0.93–2.43) for < 30 mL/min compared with eGFR of > 89 mL/min. Adjusted HR for stroke occurrence among patients with hypertension with eGFR < 60 mL/min was 3.69 (1.49–9.13) (*p* = 0.0047) and among those with diabetes was 1.50 (0.56–3.98) (*p* = 0.42).	Chronic kidney disease is dose-dependently associated with occurrence of incident of strokes among Ghanaians with hypertension and diabetes mellitus.	1. Funding for health infrastructure and availability of skilled personnel to managed CVD in Africa 2. Regular monitoring of renal function of patients with hypertension and/or diabetes mellitus may be a cost-effective strategy in SSA given the dose-response association observed between stages of CKD and risk of stroke occurrence in the present study

**Table 4:** Assessment of risk of bias of selected studies.

**Title of study**	**Representativeness of the study population**	**Reliability of data collection**	**Consistency in methodology**	**Temporal changes and confounding**
Trends in stroke admission and mortality rates from 1983 to 2013 in central Ghana	The study conducted at the Komfo Anokye Teaching Hospital (KATH) in Kumasi, Ghana, focuses on stroke admissions and mortality rates over a 30-year period. While this provides a valuable longitudinal dataset, the reliance on hospital-based data introduces a potential risk of selection bias. The patient population at a tertiary referral hospital may not be fully representative of the broader community, particularly since it excludes individuals who experienced stroke but did not seek hospital care or were treated at other facilities. This could lead to an overrepresentation of more severe cases, skewing the trends in stroke admissions and mortality rates observed in the study.	The study utilizes retrospective data collection from hospital records, which may introduce information bias due to inaccuracies in record-keeping, missing data, or changes in diagnostic criteria over time. The transition from ICD-9 to ICD-10 codes during the study period could have affected the consistency of stroke classification, further introducing variability in the data. Additionally, the absence of neuroimaging prior to 2008 means that earlier cases were categorized based on clinical assessment alone, potentially leading to misclassification of stroke subtypes.	Although the study adheres to the WHO definition of stroke for diagnosis, the lack of neuroimaging for a significant portion of the study period introduces a risk of bias. This could affect the accuracy of stroke subtype classification, with ischemic and hemorrhagic strokes potentially being misclassified. Moreover, the study mentions missing tally cards for certain years (e.g., 1989 and 1990), which could result in incomplete data and affect the reliability of trends analysis.	Over the 30-year period, there were likely significant changes in healthcare infrastructure, stroke awareness, and treatment protocols at KATH, which are not fully accounted for in the analysis. These factors could confound the observed trends in stroke admissions and mortality, leading to potential bias in interpreting the data as solely reflective of changes in stroke incidence or severity.

Incident stroke among Ghanaians with hypertension and diabetes: A multicentre prospective cohort study	The representativeness of the study population is a key strength of the study, particularly when evaluated using the WHO STEPwise approach to stroke surveillance (WHO STEPS) framework. The study's inclusion of participants from multiple hospitals across different regions in Ghana, representing a wide range of demographic, socioeconomic, and geographic characteristics, enhances the generalizability of the findings. By ensuring that the study population closely mirrors the broader population, the study provides valuable insights into stroke risk factors that are applicable at a national level. The study's adherence to the WHO STEPS framework in selecting and analyzing a representative sample strengthens the validity and relevance of its conclusions.	Using the WHO STEPwise approach to stroke surveillance (WHO STEPS) framework, the study appears to have implemented several practices that enhance the reliability of data collection. The use of standardized questionnaires, consistent physical measurements, accredited laboratory procedures, and comprehensive training for personnel are all in line with WHO STEPS recommendations and contribute to the reliability of the data. Quality assurance measures, including equipment calibration, data verification, and consistent follow-up, further ensure that the study's findings are based on reliable and accurate data.	The study demonstrates a high level of consistency in methodology by adhering closely to the WHO STEPwise approach to stroke surveillance (WHO STEPS). Through the use of standardized questionnaires, consistent physical and biochemical measurement protocols, and uniform implementation across multiple sites, the study ensured that data collection was reliable and comparable. The consistent application of these methodologies, supported by training, quality control, and the use of standardized tools and procedures, aligns with the WHO STEPS framework and enhances the overall validity of the study's findings.	The study's approach to managing temporal changes and confounding is aligned with the WHO STEPwise approach to stroke surveillance (WHO STEPS) framework. By conducting regular follow-ups, identifying and adjusting for potential confounders, and accounting for temporal changes in both risk factors and confounders, the study effectively minimizes bias and provides a robust analysis of stroke risk factors. The careful handling of these methodological aspects ensures that the study's findings are reliable, valid, and reflective of the true relationships between stroke risk factors and outcomes over time.

Estimated glomerular filtration rate predicts incident stroke among Ghanaians with diabetes and hypertension	The study made efforts to ensure representativeness through a diverse selection of hospitals and standardized data collection methods; its focus on a hospital-based population may limit the broader applicability of the results to all Ghanaians, particularly those outside the healthcare system.	The study “Estimated glomerular filtration rate predicts incident stroke among Ghanaians with diabetes and hypertension” appears to have made significant efforts to ensure the reliability of data collection, particularly through the use of standardized protocols, trained data collectors, and validated measurement tools. However, the reliability of the data could be further enhanced by documenting calibration procedures, implementing robust quality control measures, and ensuring consistency across multiple sites. By adhering to the recommendations of the WHO STEPwise approach to stroke surveillance, the study can minimize the risk of bias and ensure that its findings are accurate, consistent, and generalizable.	The study followed the WHO STEPS framework to ensure consistency in data collection and analysis. Standardized questionnaires were used to collect demographic, behavioral, and medical history data across all study sites, ensuring uniformity in the information gathered. Consistent procedures were followed for measuring height, weight, waist circumference, and blood pressure. Blood samples were analyzed in an ISO-certified lab to ensure accuracy, particularly in evaluating renal function using the estimated glomerular filtration rate (eGFR). Stroke diagnoses were standardized using the WHO definition and a validated questionnaire, with follow-up assessments conducted regularly. This ensured consistent monitoring of participants for incident strokes. Overall, adherence to the WHO STEPS framework ensured reliable and comparable data, supporting the study's findings on the relationship between renal impairment and stroke risk.	The study “Estimated glomerular filtration rate predicts incident stroke among Ghanaians with diabetes and hypertension” presents valuable findings, but it is crucial to consider temporal changes and confounding factors in interpreting these results. The WHO STEPwise approach to stroke surveillance (WHO STEPS) framework highlights the importance of adjusting for confounders, accounting for temporal trends, and considering the potential for unmeasured confounding. While the study made efforts to adjust for key confounders, the potential for residual and unmeasured confounding, as well as the relatively short follow-up period, could introduce bias into the findings. Addressing these issues through more extended follow-up and comprehensive adjustment for confounders would enhance the robustness and generalizability of the study's conclusions.

## Data Availability

The available data was presented in the main manuscript. No supplementary data was generated in this study.
